# A model of human APOA2 on HDL

**DOI:** 10.1016/j.jlr.2026.101113

**Published:** 2026-07-27

**Authors:** Yi He, Hyun Song, Youngki You, John T. Melchior, Martin K. Jones, Stephen G. Aller, W. Sean Davidson, Jay W. Heinecke, Jere P. Segrest

**Affiliations:** 1 Department of Medicine, University of Washington, Seattle, WA, USA; 2 Department of Medicine, Vanderbilt University Medical Center, Nashville, TN, USA; 3 Biological Sciences Division, Pacific Northwest National Laboratory, Richland, WA, USA; 4 Department of Pathology and Laboratory Medicine, University of Cincinnati, Cincinnati, OH, USA; 5 Department of Neurology, Oregon Health and Science University, Portland, OR, USA; 6 Department of Biochemistry and Molecular Genetics, University of Alabama at Birmingham, Birmingham, AL, USA

**Keywords:** apolipoproteins, lipoprotein metabolism, apolipoprotein A-I, apolipoprotein A-II, chemical cross-linking

## Abstract

Apolipoprotein A-II, the second most abundant protein in HDL, plays a key role in HDL maturation and reverse cholesterol transport. It has a stronger affinity for lipids than apolipoprotein A-I (APOA1), which forms HDL's structural scaffold. Like APOA1, APOA2 mainly consists of amphipathic alpha helices that help it interact with lipid surfaces. Using computer modeling and mutagenesis, we developed a structural model for lipid-bound human APOA2. The protein’s single disulfide bond restricts it to a belt-like configuration in discoidal HDL particles. Our model shows several key features: (i) most basic residues of APOA2 are in the hairpin loop; (ii) salt bridges and π-π interactions stabilize the structure; (iii) two high-lipid affinity helical regions are in each APOA2 monomer; and (iv) the hairpin structure facilitates interactions with other proteins like APOA1 or APOA2. Chemical crosslinking and MS/MS analysis identified 16 crosslinks; Fifteen of the 16 crosslinks are consistent with the double hairpin belt model, strongly supporting the proposal that the disulfide-dimer double hairpin belt is the primary structure of APOA2 in humans.

In humans, apolipoprotein A-II (APOA2) is a 77-amino acid polypeptide that circulates in plasma primarily on high-density lipoproteins (HDL) as a homodimer linked by a disulfide bond at residue 6 (1, 2, 3). APOA2 is the second most abundant protein in plasma HDL, accounting for roughly 20%–25% of the total protein mass (4). APOA2 is present on more than two-thirds of all circulating HDL. Like apolipoprotein A-I (APOA1), the primary scaffold protein of HDL, APOA2 contains a tandem array of amphipathic helices that mediate lipid binding (5, 6, 7). Although classified as an exchangeable apolipoprotein, APOA2 displays substantially higher lipid affinity than APOA1 (7), suggesting limited dynamics once associated with particles in plasma.

Clinical studies show APOA2 levels are inversely associated with cardiovascular disease (CVD) risk (8, 9, 10), yet the mechanisms underlying this apparent protection remain unclear. Given HDL’s well-documented role in reverse cholesterol transport and that most HDL contain APOA2, one proposed function is that APOA2 enhances cholesterol efflux capacity. Consistent with this notion, APOA2 levels are strongly and positively associated with cholesterol efflux capacity (11), which predicts CVD risk independently of HDL cholesterol (HDL-C) and APOA1 levels (12). Moreover, the presence of APOA2 on both reconstituted HDL discs and native plasma HDL enhances cholesterol efflux capacity compared to their counterparts containing just APOA1 (13). More recently, APOA2 was shown to enhance ABCA1-mediated cholesterol efflux through modulation of the APOA1 C-terminus and to induce conformational changes in the N- and C-terminal regions of APOA1, providing a potential mechanism for the enhanced cholesterol efflux (14).

Despite its central role in lipid metabolism and cardioprotection (8, 9, 10, 11, 15, 16, 17, 18), its structural organization on HDL and interactions with APOA1 remain poorly defined. Biophysical analyses of reconstituted HDL discs containing only APOA2 suggest it forms a homodimeric, antiparallel belt-like structure in which helices wrap around the phospholipid bilayer (19). Hydrogen-deuterium exchange data indicate that APOA2 interacts with helical repeats 3-4 and 8 of APOA1 on reconstituted HDL (rHDL) (20). Limited proteolysis-mass spectrometry experiments generally agree in showing that APOA1 helical regions 7-8 are masked from digestion after addition of APOA2. However, despite insights from these studies, a comprehensive, experimentally supported structural model of APOA2 on APOA1-containing lipid particles, is still lacking.

Herein, we combined computational modeling, site-directed mutagenesis, and chemical cross-linking-mass spectrometry (XL-MS) to define the structural arrangement of APOA2 and APOA1 on lipid particles. We carried out detailed structural analysis of homogenous rHDL containing both APOA1 and APOA2 and compared features of our model to data acquired on native, human HDL isolated from plasma. Our resulting model provides a framework for understanding how APOA2 shapes native HDL particle structure and function and provides insights into potential mechanisms by which it may confer cardioprotective effects in humans.

## Materials and methods

### Materials

Human APOA1 and APOA2 were purchased from Academy Bio-Medical (Houston, TX). Palmitoyl-oleoyl-phosphatidylcholine (POPC) was purchased from Avanti Polar Lipids. Free cholesterol and Deoxycholic acid sodium salts were purchased from Sigma-Aldrich. Cross-linking reagent 1-ethyl-3-(3-dimethylaminopropyl)carbodiimide hydrochloride (EDC) was purchased from ThermoFisher scientific.

### Generation of A-I/A-II rHDL

To understand the orientation of APOA2 on HDL, we generated reconstituted HDL discs (rHDL) that contain both APOA1 and APOA2 (A-I/A-II rHDL) as previously described (13, 20, 21, 22). Particles were generated in two independent laboratories using either the Bio-Bead method or cholate dialysis method (20, 21, 22). In both cases, the A-I/A-II rHDL was prepared using a ratio of 160: 2:1 (POPC: APOA1: APOA2). Sizes of the rHDL particles were determined using calibrated ion mobility analysis (IMA). A-I/A-II-POPC-rHDL particles demonstrated a homogeneous distribution with average size of 9.9 ± 0.1 nm.

### Isolation of human plasma HDL

Plasma was prepared from EDTA-anticoagulated blood collected from healthy adult subjects who had fasted overnight. HDL (density 1.125–1.210 g/ml) was isolated from plasma by sequential ultracentrifugation, snap frozen and stored −80°C until use. Upon thaw, the HDL was further purified by size exclusion chromatography (Superdex 200, 350 μl flow/min) and fractions corresponding to the purest HDL peak collected.

### Ion mobility analysis of plasma and rHDL particles

HDL was analyzed using a scanning mobility particle sizer spectrometer (TSI Inc., Shoreview, MN, model 3080N) fitted with a nano-differential mobility analyzer (TSI Inc., model 3085) and a charge-reducing electrospray ionization source (CR-ESI; TSI Inc., model 3480). Monodisperse particles exiting the differential mobility analyzer were detected by a condensation particle counter (TSI Inc., model 3788). Samples were introduced into the electrospray chamber every 15 min by automated loop injections. Particle concentration was determined by external calibration (23).

### Chemical cross-linking of plasma and rHDL particles

To investigate the structural conformations of APOA2, we generated reconstituted HDL particles (rHDL) containing both proteins and performed chemical cross-linking. Three separate cross-linking reagents that react with varied amino acids and have different spacer arm lengths were used to maximize resolution of amino acid proximity on the particles: 1-ethyl-3-(3-dimethylaminopropyl)carbodiimide hydrochloride (EDC), bis-(sulfosuccinimidyl)suberate (BS3) (Thermo Scientific), and disuccinimidyl sulfoxide (DSSO) (Thermo Scientific). EDC and BS3 reactions were carried out in phosphate-buffered saline (PBS, pH 6.5). EDC was added at a molar ratio of 1000:1 EDC to APOA1, while BS3 was added at a molar ratio of 50:1 BS3 to APOA1 for the rHDL particles. Plasma HDL samples were cross-linked with BS3 and DSSO at a final protein concentration between 0.5 and 0.75 mg/ml. DSSO reaction was performed in 50 mM HEPES buffer. Reactions for both EDC and BS3 cross-linking experiments were allowed to take place for 12 h at 4°C. DSSO cross-linking was also performed for 1 h at room temperature. Reactions were quenched by bringing the final concentration of samples to 20 or 50 mM with standard Tris buffer. To eliminate rare instances of rHDL particles being linked together during the cross-linking reaction, reaction mixtures were further fractionated by high-resolution size-exclusion chromatography to isolate single, cross-linked rHDL. Fractions corresponding to the purified cross-linked HDL were pooled and stored at 4°C until ready for LC-MS/MS analysis.

### Sample preparation for LC-MS/MS analysis

EDC crosslinked plasma HDL and BS3 crosslinked rHDL samples were exchanged into 50 mM NH_4_HCO_3_ (pH = 8.1) and subsequently lyophilized to dryness. To isolate protein and remove lipid from samples, we performed an organic solvent extraction using chloroform: methanol (2:1 v/v) as previously described (13). The solubilized, cross-linked proteins were reduced using 10 mm DTT at 42°C for 30 min and free cysteines alkylated using 40 mM iodoacetamide at room temperature in the dark for 30 min. Cross-linked proteins were digested by addition of sequencing-grade trypsin (Promega, Madison, WI) at a ratio of 20:1 (w/w) protein/trypsin and incubated at 37°C overnight. Digestion was halted by acidification (pH 2–3) with trifluoroacetic acid. BS3- or DSSO-crosslinked plasma HDL samples were denatured with 8 M urea. After denaturation, proteins were reduced with 5 mM DTT for 1 h at 25°C, followed by alkylation with 40 mM iodoacetamide for 45 min at 25°C in the dark. Samples were diluted threefold with 50 mM ammonium bicarbonate and digested with trypsin at an enzyme: substrate ratio of 1:50 (w/w) for 16 h at 25°C. Protease activity was quenched by adding 0.1% formic acid.

### LC-MS/MS peptide analysis

Capillary LC-ESI-MS/MS was performed using an IntegraFrit capillary C18 trapping column (Waters XBridge BEH C18, 5 μm, 0.1 × 20 mm), a capillary analytical C18 column packed in-house (Waters XBridge BEH C18, 5 μm, 0.075 × 150 mm) and an LTQ Orbitrap XL mass spectrometer (Thermo Electron). The nanoACQUITY UPLC (Waters) was used for the separation, with a linear gradient of 0.1% formic acid in water (solvent A) and 0.1% formic acid in acetonitrile (solvent B). In each experiment, 1 μg of tryptic digest was injected onto a trapping column to remove salts and contaminants. Effluent from the trapping column was directed to the capillary analytical column with an 80-min gradient between 2% and 40% mobile phase B at a flow rate of 0.3 μl/min. Eluting peptides were electrosprayed into an LTQ-Orbitrap mass spectrometer operating in the data-dependent mode to acquire a full MS scan (400–2000 m/z) and then subsequent MS/MS scans of the five most intense precursor ions. Isolation width was 2 m/z. MS/MS scans were acquired in the ion trap. Collision-induced dissociation (CID) was performed at 35% normalized collision energy. Charge state rejection was enabled for 1+ and 2+ charge states. As cross-linked peptides formed in these experiments tend to have higher charge states upon ESI than non-cross-linked (linear peptide) species, rejection of low-charge states from MS/MS acquisition enhances detection of crosslinks. For database searching, MS/MS spectrum lists were extracted from the raw files and converted to mzXML files, using ReAdW (version 4.6.0).

Cross-linked peptides of plasma HDL from BS3 and DSSO were analyzed on an Orbitrap Fusion Lumos mass spectrometer. Peptides were reconstituted in 0.1% formic acid and separated on a Thermo Vanquish Neo LC with an in-house packed Waters AQ C18 analytical column (30 cm × 75 μm i.d., 1.7 μm). Chromatography was performed at 0.2 μl/min using mobile phase A (0.1% formic acid in water) and mobile phase B (0.1% formic acid in acetonitrile). Peptides were eluted over 120 min using a detailed gradient. MS1 spectra were acquired from 375–1600 m/z at a resolution of 120K. MS/MS spectra were acquired in data-dependent mode using a 1.6 m/z isolation window. Peptides were fragmented by CID at a normalized collision energy of 20%, 25%, and 30%, with a 60 s dynamic exclusion, analyzing charge states +4 to +8 at the MS1 level and +2 to +6 at the MS/MS level. For MS^3^, up to four MS^3^ scans were triggered per MS^2^ event and acquired in the ion trap using quadrupole isolation (2.5 m/z window) and HCD fragmentation at a normalized collision energy of 35%.

### Cross-linking data analysis

Crosslinking data analysis was performed using the Spectrum Identification Machine for Cross-linked Peptides (SIM-XL, version 1.5.6.1), as previously described (24). Crosslinks were identified by comparing experimental mass spectra from cross-linked peptides to theoretical spectra from the reference database. Assignments are made when experimental and theoretical precursor and MS/MS spectra are matched. SIM-XL files were searched against a custom FASTA file containing sequences for human APOA1, APOA2, and serum albumin. Serum albumin served as a negative control to establish a scoring threshold for positive identifications and to rule out false cross-links. Positive identifications have less than 5% false-discovery rate (FDR) based on a target-decoy calculation strategy (a reverse decoy database). For DSSO crosslinked peptides, cross-link spectrum matches were identified using XlinkX with Proteome Discover (version 3.2) with the FDR set to 1%. In all cases we manually inspected all identified cross-links to ensure accuracy: the cross-linked product had been sequenced and that most of the abundant fragment ions could be assigned in the MS/MS spectra.

### APOA2 model generation

The lipid affinity (termed Λ4) of human APOA1 and APOA2 for each helical domain were calculated using the WHEEL algorithm (5, 25, 26), with two distinct helical pitches: the idealized 18/5 and the proposed 11/3. The LOCATE algorithm (5, 25, 26, 27) identified two very strongly amphipathic α-helical domains in APOA2, residues 5–31 and 51–73; the residue 32–50 domain was identified as very weakly amphipathic. Helical wheel diagrams of the two strongly amphipathic domains of APOA2 were generated using the WHEEL subroutine.

To construct the atomic structure of monomeric human APOA2, which consists of 77 amino acids, the Build plugin in PyMOL was used. The second domain was specifically modeled to form a curved hairpin conformation, thereby separating two highly amphipathic helical domains. This structural arrangement was modeled based on experimental chemical cross-linking data, with the resulting structure supporting fifteen of the sixteen identified crosslinks.

For the dimeric structure, a disulfide bond was formed between the C6 residues in an antiparallel orientation, positioning the hydrophobic faces of the domains in opposite directions. The resulting dimer was energy-minimized using the conjugate gradient method in NAMD 2.13b2 (28) with the CHARMM36 force field (29) for 10,000 steps to eliminate potential atomic clashes. All structural figures of APOA2 were rendered using PyMOL version 3.1.1 (30).

To generate the APOA1–APOA2 interaction model, the constructed APOA2 dimer was docked onto the previously simulated small spherical HDL particle (31). The central region of the APOA2 dimer was bent to fit the curvature of the spherical HDL surface. Given the high mobility of the APOA1 C-terminus, its position was adjusted to satisfy the experimentally determined cross-links.

Based on our previous work, 50 POPC molecules were used to represent the lipid composition of an ∼80 Å spherical HDL particle (32). In addition, 20 cholesteryl ester molecules were included, corresponding to approximately 30 mol% of total lipid, consistent with reported HDL lipid composition (33). Our model consists of two APOA1 molecules and one APOA2 dimer, consistent with reported LpA-I/A-II stoichiometry (34). The system was subsequently subject to energy minimization using the same protocol described above.

Input FASTA sequences of 18 mammalian APOA2 orthologs were obtained from the NCBI website (https://www.ncbi.nlm.nih.gov/protein/), and sequence alignment was performed using CLUSTAL Omega (version 1.2.4) (35). Our APOA2 monomer model was compared with the APOA2 model generated by the AlphaFold Server (https://alphafoldserver.com), which is powered by AlphaFold3 (36), and with an APOA2 model generated by RoseTTAFold2 (37). RoseTTAFold2 was downloaded from GitHub and installed locally, and the model was generated using the Cheaha Supercomputer. We note that only monomer models were generated because these methods do not account for intermolecular disulfide bond formation during dimer modeling. For the AlphaFold3 model, the monomer was extracted from the predicted dimeric structure, which exhibited a more compact conformation and better satisfied the crosslinking restraints.

## Results

### Identification of amphipathic helical domains and lipid affinities in APOA2

**Fig. 1:**
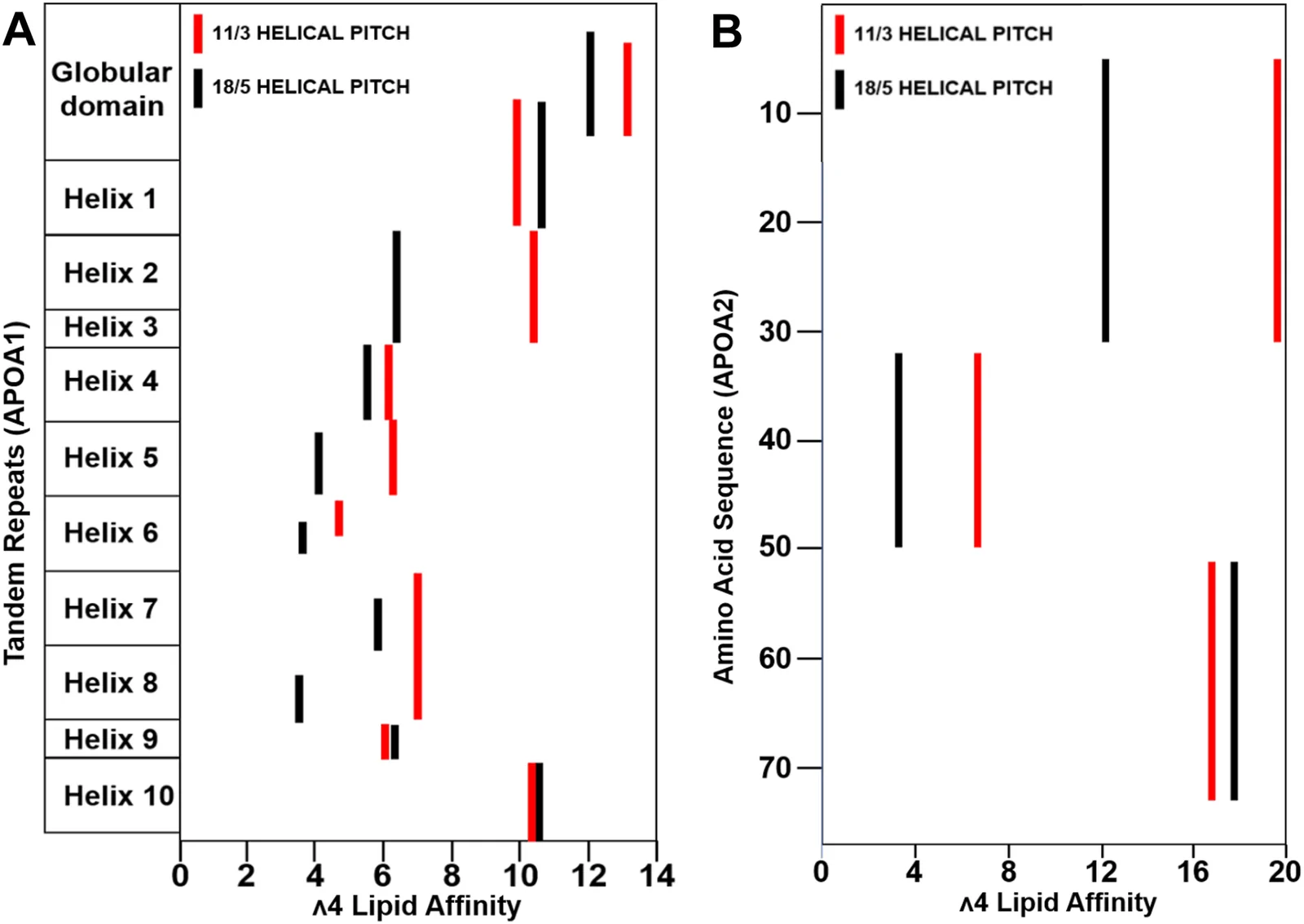
Comparison of the amphipathicity of human APOA1 and APOA2 measured with the LOCATE algorithm (25) using two different helical pitches: the idealized α-helical pitch (18/5 in black) and the proposed pitch (11/3 in red) for the APOA1 double belt structure in discoidal HDL (26). Amphipathic α-helixes are the most important surface lipid-associating motifs in exchangeable plasma lipoproteins. The sequence extent of each amphipathic helix is denoted on the y-axis by a vertical bar whose Λ4 lipid affinity corresponds to its position on the x-axis. A: LOCATE analysis of human APOA1. B: LOCATE analysis of human APOA2.

Amphipathic α-helices provide readily reversible lipid association, act as peptide detergents, and have optimal interactions with phospholipid bilayers. As the first step in developing a lipid-associating structure for APOA2, human APOA2 was analyzed by our computer algorithm for amphipathic helices, LOCATE, and the results compared with APOA1 (Fig. 1). In APOA1, there are eight 22-mer and two 11-mer tandem amino acid sequence repeats (y-axis, Fig. 1A), each with the periodicity of an amphipathic α-helix (38, 39), encoded in exon 4 of the APOA1 gene (residues 44–241). In agreement with our previous study (26), the amphipathic helical domains 1–10 in APOA1 form a straight (planar) hydrophobic face when adjusted to an α11/3 pitch but form one complete turn of a right-handed spiral when adjusted to the ideal α18/5 pitch. It therefore is not surprising that essentially all of the amphipathic helical domains in APOA1 have higher lipid affinities analyzed with the α11/3 pitch algorithm than when analyzed with the ideal α18/5 pitch algorithm (25, 26); the highest Λ4-lipid affinities are located in the N- and C-terminal domains of APOA1 (Fig. 1A).

**Fig. 2:**
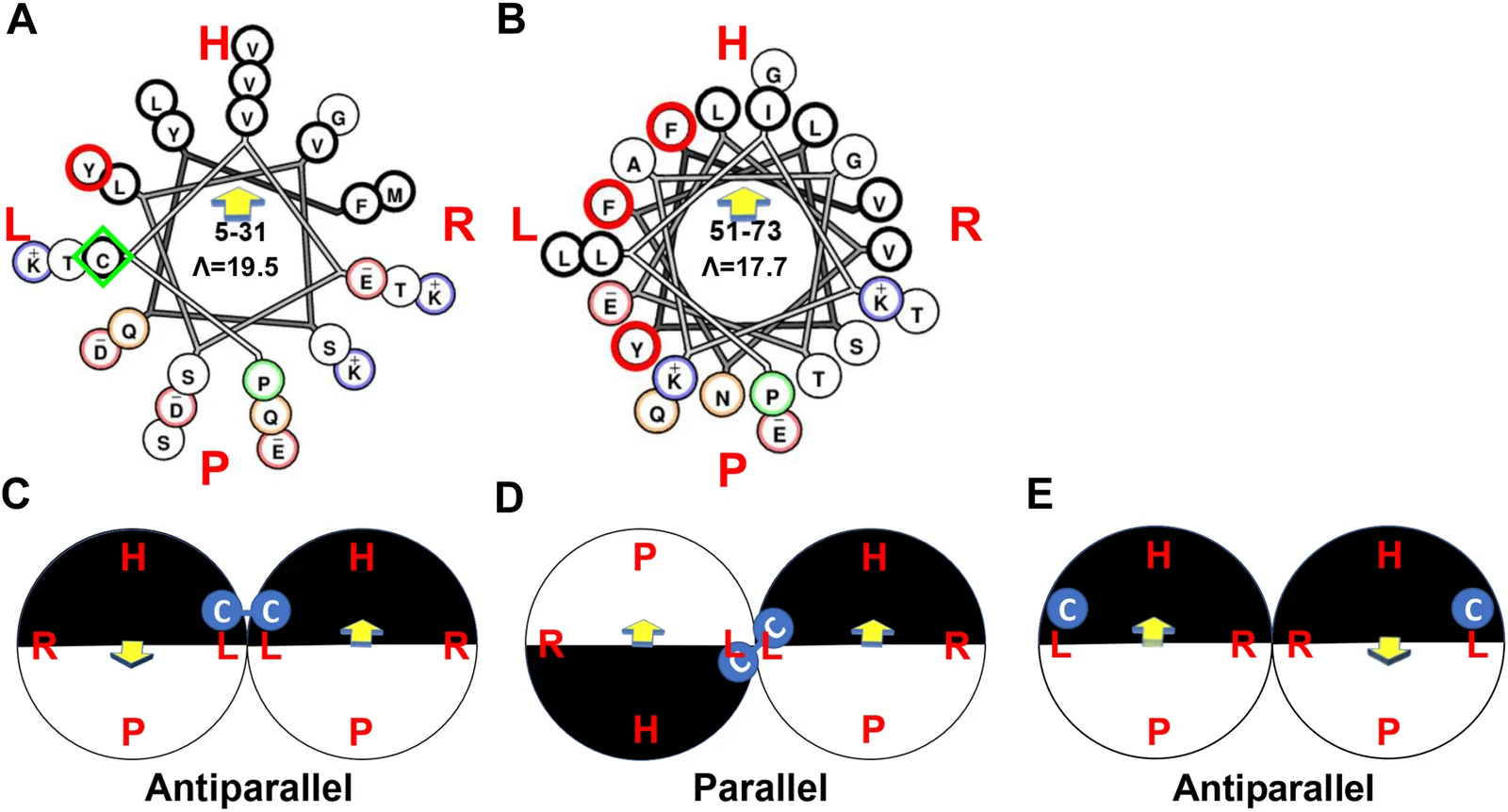
Analyses of the amphipathicity of human APOA2 with the program LOCATE. A, B: Helical wheel domains of the first (residues 5–31) and last (residues 51–73) amphipathic helical domains, respectively, identified by the program LOCATE in Figure 1 are plotted with the WHEEL subroutine. A, B: The helix in panel A is constructed with an α11/3 pitch since that gives a higher Λ4 lipid affinity than the standard α18/5 algorithm (25, 26); the panel B helix is constructed with the idealized 18/5 pitch. In each representation, the helix is oriented so that the N-termini points out of the page (yellow arrows). By definition, that defines the left (L) and right (R) amphipathic helical edges. The Λ4 lipid affinity calculated for each domain is indicated within each helical wheel. The four aromatic residues involved in the proposed π-cup (see Fig. 4B) are indicated by red circles. Residue C6 is indicated by a green diamond in panel A. C, D: Cartoon views of the two helical orientations allowing formation of a disulfide bond, creating, respectively, antiparallel and parallel helical pairings. Note that parallel pairing places hydrophobic faces (H) in opposite directions. Here, (P) denotes the polar faces. E: Cartoon representation of the open double hairpin model. Although this model adopts an antiparallel configuration, the RR lines of contact position the two Cys6 residues on opposite sides of the dimer, thereby preventing disulfide bond formation between the two APOA2 monomers.

Analysis of APOA2 identifies three amphipathic α-helical domains (Fig. 1B), residues 5–31 (AH1), 32–50 (AH2) and 51–73 (AH3). The middle domain has a low Λ4-lipid affinity, but the two terminal domains exhibit Λ4-lipid affinities significantly higher than the maximum values (10–13) observed in APOA1. The N-terminal domain, residues 5–31, has a Λ4-lipid affinity of almost 20 by the α11/3 pitch algorithm (Fig. 2A); the C-terminal domain, residues 51–73, has a Λ4-lipid affinity ranging from 17–18 (Fig. 2B) that varies little between helical pitches. These two amphipathic helices have a summed Λ4-lipid affinity of approximately 37, potentially explaining why APOA2 is almost exclusively bound to lipid in the plasma (40).

We performed multiple sequence alignment of APOA2 orthologs from 18 mammalian species using CLUSTAL Omega (Supplemental Fig. S1). The alignment revealed differential conservation among the four proline residues in human APOA2 (P5, P32, P51, and P74). While P5 and P32 are not conserved across species, P51 and P74 are fully conserved. Overall, 51 of the 77 residues (∼66%) in human APOA2 are either completely or strongly conserved. Conservation levels vary by domain, with 74%, 84%, and 48% conservation for the AH1, AH2, and AH3 regions, respectively.

We also observed sequence variation in the C-terminus of APOA2 (Supplemental Fig. S1). Among the final four residues (PATQ) in human APOA2, the proline (P) is completely conserved, alanine (A) is strongly conserved, while threonine (T) and glutamine (Q) show no conservation. This variation suggests the potential existence of APOA2 isoforms. Indeed, a previous study by Honda *et al.* identified five circulating isoforms of APOA2 in human plasma, differentiated by truncations at the C-terminus (41).

Together, these analyses identify two α-helical domains (AH1 and AH3) with high lipid affinity at the N- and C-termini, providing a structural basis for a hairpin-loop (AH2)–containing APOA2 structure used for subsequent model generation.

### Generation of APOA2 dimer models

To obtain distance restraints to guide initial models of APOA2, we performed XL-MS on medium-sized rHDL containing both APOA1 and APOA2. Particle diameters were first determined using calibrated ion mobility revealing an average diameter of 100 Å (Supplemental Fig. S2). We used three complementary cross-linkers: EDC, a zero-length reagent that forms amide bonds primarily between carboxylate- and amine-containing residues, BS3, a longer, lysine-reactive NHS-ester, and DSSO. Using these three cross-linking strategies, we identified six Glu-Lys and ten Lys-Lys intramolecular chemical crosslinks within APOA2 (Table 1). Each experiment included three technical replicates, and cross-link confidence was ranked based on reproducibility; cross-links observed in a single replicate were classified as weak, whereas those detected in all three replicates were considered strong. We note that six intramolecular cross-links identified in this study (residues 39–44, 44–46, 30–44, 30–46, 30–54, and 39–46) were also reported in a previous study using rHDL (19). Importantly, these cross-links were independently observed in our XL-MS experiments, thereby providing additional experimental support for the APOA2 structural model.

**Table 1:** Intra-molecular distances between cross-linked residues of APOA2 identified using EDC (zero-order), DSSO, and BS3 in plasma UC-HDL, and BS3 in ∼100 Å discoidal rHDL

Type of HDL	Crosslink type	Residues	Relative intensity	Cα–Cα distance (Å), Match		
				Our model	AlphaFold3	RoseTTAFold2
UC-HDL	Zero-Order Crosslinks	E8-K46	Weak	48.6 (N)	**14.4** (Y)	47.0 (N)
		K30-E33	Weak	**4.8** (Y)	**8.7** (Y)	**8.9** (Y)
		E33-K44	Strong	**15.9** (Y)	16.6 (N)	16.6 (N)
		E37-K39	Weak	**5.4** (Y)	**5.4** (Y)	**5.2** (Y)
		E37-K46	Weak	**15.3** (Y)	**14.5** (Y)	**13.9** (Y)
		K39-E47	Medium	**9.4** (Y)	**12.6** (Y)	**12.2** (Y)
	DSSO Crosslinks	K23-K30	Medium	**11.6** (Y)	**9.3** (Y)	**10.2** (Y)
		K30-K44	Medium	**18.8** (Y)	**20.7** (Y)	**19.1** (Y)
		K30-K54	Medium	**15.9** (Y)	35.4 (N)	31.4 (N)
		K30-K55	Medium	**12.7** (Y)	36.3 (N)	33.2 (N)
		K39-K46	Medium	**12.4** (Y)	**11.2** (Y)	**10.1** (Y)
	BS3 Crosslinks	K23-K30	Strong	**11.6** (Y)	**9.3** (Y)	**10.2** (Y)
		K30-K46	Medium	**20.3** (Y)	**23.0** (Y)	**19.7** (Y)
		K39-K46	Strong	**12.4** (Y)	**11.2** (Y)	**10.1** (Y)
rHDL	BS3 Crosslinks	K23-K28	Weak	**8.7** (Y)	**8.2** (Y)	**8.5** (Y)
		K23-K30	Weak	**11.6** (Y)	**9.3** (Y)	**10.2** (Y)
		K23-K55	Strong	**16.6** (Y)	29.5 (N)	39.2 (N)
		K30-K55	Medium	**12.7** (Y)	36.3 (N)	33.2 (N)
		K39-K44	Strong	**8.4** (Y)	**8.7** (Y)	**8.5** (Y)
		K39-K46	Strong	**12.4** (Y)	**11.2** (Y)	**10.1** (Y)
		K44-K46	Weak	**5.9** (Y)	**5.6** (Y)	**5.2** (Y)

Intra-molecular Cα–Cα distances were measured from our model, AlphaFold3, and RoseTTAFold2. Distances below than the cutoff for zero-order crosslinks (nominally 15 Å, but here adjusted to 16 Å to allow small variation) and for BS3 and DSSO (25 Å) are shown in bold. Agreement with the experimental cross-linking data is indicated as Y (yes) or N (no). The fourth column lists the relative peak intensity of MS/MS signals.

By comparing Lys–Lys intramolecular cross-links obtained using BS3 and DSSO on ultracentrifugation-isolated HDL (UC-HDL) with BS3 cross-links measured on rHDL (Table 1), we identified several conserved APOA2–APOA2 intramolecular crosslinks across three cross-linking strategies. Two crosslink pairs (K23–K30 and K39–K46) were consistently observed in all three datasets, and the K30–K55 crosslink was detected both in BS3-cross-linked rHDL and DSSO-cross-linked UC-HDL. Notably, the K39–K46 crosslink—one of the most reproducible restraints in the rHDL dataset—is conserved between rHDL and native UC-HDL, providing strong support for the biological relevance of the proposed APOA2 hairpin belt structure.

**Fig. 3:**
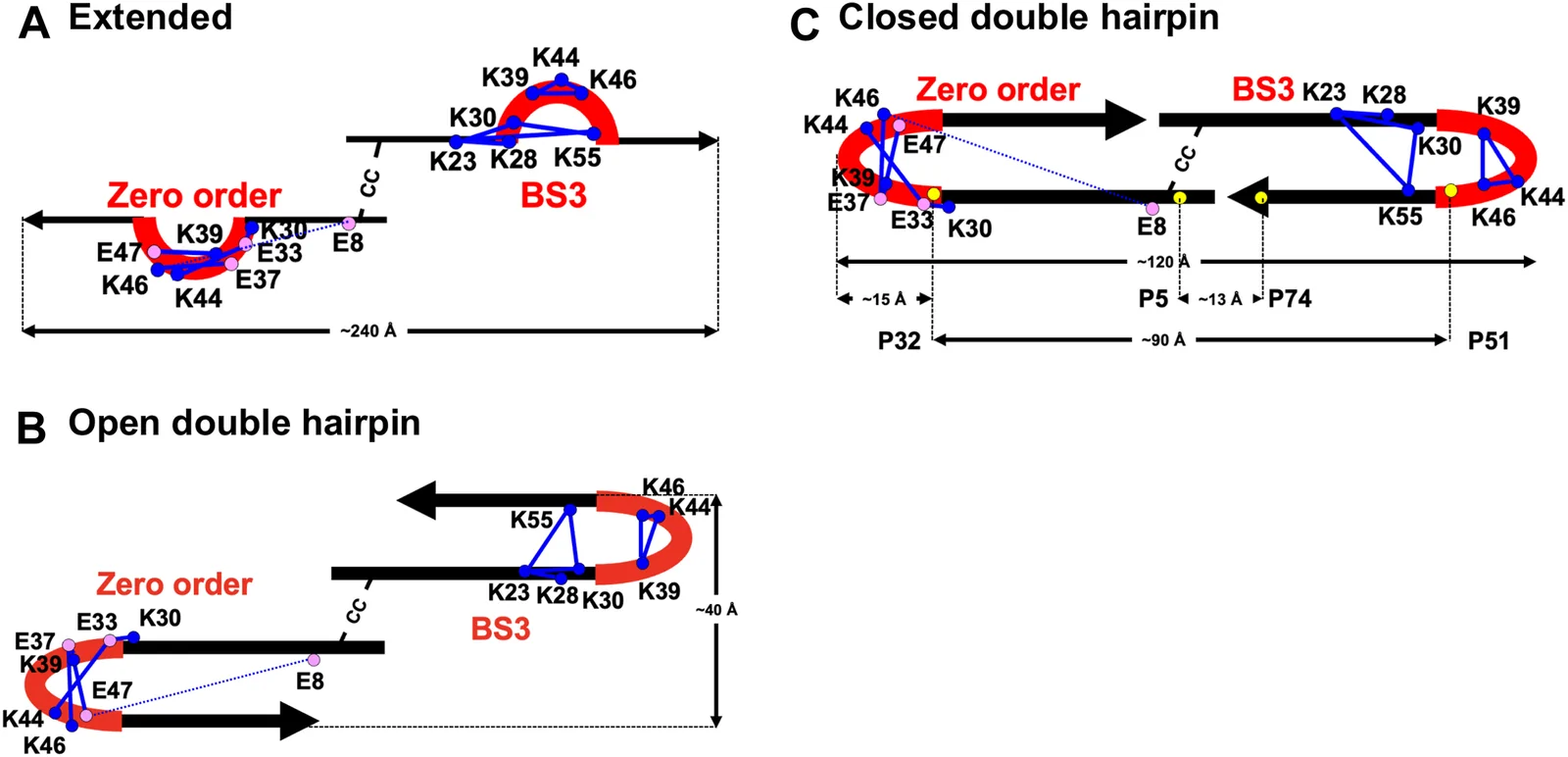
Mapping of chemical crosslinks onto two possible antiparallel disulfide-bond orientations denoted by cartoon representations. Since the parallel disulfide bond configuration forces the hydrophobic faces to face in opposite directions, the disulfide bond in human APOA2 restricts lipid-associated dimeric APOA2 to the antiparallel conformation. There are three possible conformations for the antiparallel alignment: A: An extended disulfide-linked dimer. B: An open disulfide-linked double hairpin belt. C: A closed disulfide-linked double hairpin belt. Intramolecular crosslinks determined by two different chemical methods are plotted onto the schematic diagrams (BS3 in rHDL and zero-order in UC-HDL onto opposite ends of each diagram) in panels A–C. Only representative crosslinks from Table 1 are shown for clarity. Charged residue positions are denoted by circles (basic, blue; acidic, pink). Crosslinks are denoted by solid blue lines (acceptable) and dotted blue lines (unacceptable); thick lines indicate definite crosslinks, thin lines uncertain crosslinks. Key linear dimensions are indicated for structures in panels A and C. Small filled circles colored in blue and pink indicate lysine and glutamate residues participating in experimentally observed crosslink pairs, whereas yellow circles represent the four proline residues (P5, P32, P51, and P74).

Guided by these cross-linking data, we focused on model generation. The cross-linking data strongly supported the presence of a hairpin centered around residues 32–50 that separates two highly amphipathic domains (residues 5–31 and 51–73). Given that a single amphipathic helical belt alone cannot stabilize the edge of a phospholipid disc (26, 42, 43) and that APOA2 alone can stabilize phospholipid into their own discoidal rHDL (19), we reasoned that APOA2 is likely forming a ∼20 Å thick, antiparallel structure similar to APOA1. We therefore derived three possible antiparallel dimer arrangements compatible with a disulfide-linked homodimer containing hairpins guided by cross-links, which are displayed in Figure 3: (i) an extended disulfide-linked dimer (Fig. 3A); (ii) an open disulfide-linked double hairpin belt (Fig. 3B); and (iii) a closed disulfide-linked double hairpin (Fig. 3C). In all three cases, fifteen of the sixteen cross-links fit the models and strongly support the double hairpin belt model (Table 1). Importantly, E33-K44, K39-E47, K39-K44, and K39-K46 are the four crosslinks with high intensity, indicating the double hairpin belt is the major conformation of the APOA2 dimer. Only one weak crosslink (E8-K46) is not compatible with our model, suggesting there may be a different structural orientation present in low abundance in small human HDL.

### Evaluation of APOA2 dimer models

Unfortunately, the EDC and BS3 cross-links themselves are unable to distinguish among these three topologies. However, the extended disulfide-linked dimer model (Fig. 3A) places a hairpin turn at residues 32–50 in the middle of a long, continuous double belt. On a rHDL disc, as we have here, this configuration offers no clear structural or functional advantage. Furthermore, it has previously been reported that the parallel disulfide bond configuration in human APOA2 forces the hydrophobic faces of the two monomers to point in opposite directions, restricting the dimer to an antiparallel orientation (19). Collectively, this prompted us to eliminate the extended model given due to its low probability for existence and to focus our efforts on the double hairpin belt models. Our proposed closed double hairpin model is similar to the “double hairpin” structural model previously suggested by Silva *et al.* for APOA2 on the edge of a discoidal rHDL particle (19).

**Fig. 4:**
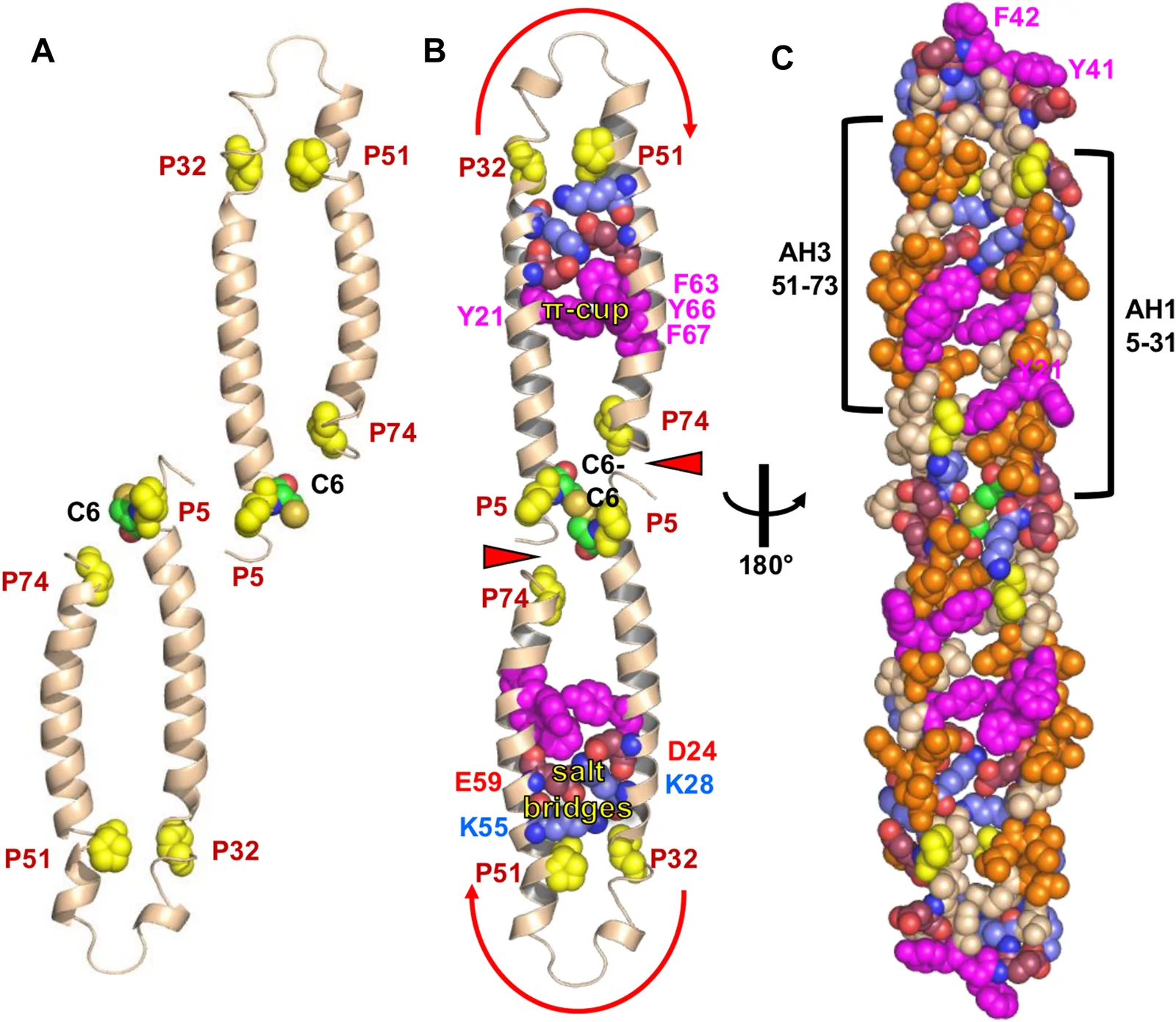
Detailed HDL-associated structures of two orientations of disulfide-linked double hairpin model of human APOA2. A: Open double hairpin model in an antiparallel arrangement shown in cartoon representation. The four proline residues are displayed as yellow space-filling spheres. The two Cys6 residues (space-filling, colored by atom type) are located on opposite sides of the model and therefore cannot form a disulfide bond. B: Energy-minimized model of the closed disulfide-linked double hairpin belt structure shown in cartoon representation. Curved red arrows mark the two hairpin loops. Red arrowheads indicate van der Waals contacts between N- and C-termini created in construction of the model. The location of the disulfide crosslink (space-filling green) and the 4 prolines (space-filling yellow) are denoted. Note the bilateral clustering of antiparallel prolines. The π−cup (Y21 nestled cross hairpin into the F63, Y66, and F67 “cup”) is marked in the upper half, as are the proposed salt bridges (residues D24, K28, K55 and E59) in the lower half. C: Structure in panel B in space-filling mode rotated 180° around the y-axis to expose the hydrophobic face. Hydrophobic residues, orange; aromatic residues, magenta; basic residues, blue; acidic residues, red; prolines, yellow; C6-C6 disulfide bond, green; all other residues, wheat. Opposed amphipathic helices, black brackets.

To examine these possible configurations in full atomic detail, we constructed all-atom models of both the disulfide-dimer double hairpin belts of APOA2 (Fig. 4). For the open double hairpin model, the two APOA2 monomers were arranged in an antiparallel orientation with their N-termini in contact. For the closed configuration, we positioned the hairpin loops to produce van der Waals contact between the N- and C-terminal ends. Energy minimization was performed on both structures prior to analysis.

We also examined helical orientations that allow disulfide bond formation between the Cys6 residues of APOA2 monomers. The left and right faces are designated L and R, respectively, when the hydrophobic face is oriented upward, the polar face downward, and the N-terminus points out of the page (Fig. 2A, B). In the LL interface, the Cys6 disulfide bond is compatible with an antiparallel arrangement in which the two monomers orient in opposite directions (Fig. 2C), consistent with the antiparallel APOA2 dimer configuration reported previously (19).

Among the open and closed double hairpin possibilities, the open disulfide-linked double hairpin model forms a 40 Å wide quadruple belt (Figs. 3B and 4A), too thick to stably cap the edge of a discoidal HDL particle. Further, we found the open hairpin configuration produces RR lines of contact between opposing amphipathic helices (Fig. 2E), and places the two Cys6 residues on opposite sides, thereby preventing disulfide bond formation, collectively suggesting this model is unstable.

By contrast, the closed double hairpin (Figs. 3C and 4B, C) yields ∼20 Å-wide double belt with LL lines of contact, which are ideally suited for stabilizing a discoidal HDL. The van der Waals contact between N- and C-terminal ends (Fig. 4B, red arrowheads) minimizes exposure of underlying fatty acyl chains compared to the open model, making the closed configuration more energetically favorable. This packing further revealed several striking structural features.

i. Two antiparallel, high lipid-affinity amphipathic helices (residues 5–31 and 51–73) form the double belt in each APOA2 monomer;
ii. The hairpin of each monomer lies halfway between the pair of amphipathic helical domains;
iii. A cluster of cross-double-belt salt bridges (D24, K28, E59, K55) occur at the base of each hairpin loop;
iv. A cluster of four aromatic residues (Y21, F63, Y66, F67) forming a π-π stacking “π-cup” immediately beneath the salt bridge cluster;
v. Two aromatics at each hairpin turn (Y41 and F42) forming a hydrophobic “corner” that could provide an interaction site for other proteins, such as APOA1 or additional APOA2 dimers; and
vi. A local clustering of basic residues in and near the loop region.

To further evaluate our model, we compared it with APOA2 monomer models generated by AlphaFold3 and RoseTTAFold2 (Supplemental Fig. S3). All three models agree that the first and third domains form amphipathic α-helices. The principal difference lies in the central domain; our model features a curved hairpin encompassing residues 32–50, whereas AlphaFold predicts a short turn around residues 29–31 and RoseTTAFold2 predicts a short turn around residues 30–32, followed in both cases by a relatively straight amphipathic α-helix. In the AlphaFold3 model, the first and third helical domains adopt an antiparallel orientation similar to our model. In contrast, the RoseTTAFold2 model adopts a substantially different conformation, with an interdomain angle greater than 90°.

We next compared the three structural models with the intramolecular cross-link pairs measured using zero-order and BS3/DSSO cross-linkers (Table 1). Our model satisfied all 10 strong- and medium-intensity cross-links, whereas the models generated by both AlphaFold3 and RoseTTAFold2 satisfied only six. The largest discrepancies involved three cross-links (K30–K54, K30–K55, and K23–K55; red lines in Supplemental Fig. S3B, C), for which the Cα–Cα distances in both the AlphaFold3 and RoseTTAFold2 models were ≥ 30 Å.

Although the current AlphaFold3 model can predict multimers, ligands, nucleic acids, and ions (36), it does not explicitly enforce covalent disulfide bond formation or account for lipid–protein interactions, which are critical for amphipathic apolipoprotein structure. Similarly, RoseTTAFold2 does not explicitly model the intermolecular Cys6–Cys6 disulfide bond. Moreover, the APOA2 models generated by either AlphaFold3 or RoseTTAFold2 do not favor the formation of the Cys6–Cys6 disulfide bond in an antiparallel configuration, which is a key structural feature driving the planar dimeric arrangement in our closed double hairpin belt.

Taken together, the convergence of the cross-linking data with the unique salt-bridge cluster, π-cup, hairpin geometry, and amphipathic double-belt organization strongly support the closed double hairpin belt model as the likely configuration of APOA2 dimer. This structure, in which the disulfide bond anchors the center and π-π and salt-bridge interactions reinforce the ends, produces a rigid, double amphipathic helical rod that can account of the exceptionally high lipid affinity of APOA2.

### Structural model of an APOA2 dimer bound to small spherical HDL containing APOA1

To explore how APOA2 dimer associates with native HDL, we next positioned the closed, disulfide-linked double hairpin belt onto small, spherical HDL that also contains APOA1. First, we generated a conceptual model in which the APOA2 dimer bends to match the curvature of an 80 Å HDL particle, with its hydrophobic face directed toward the lipid surface (Supplemental Fig. S4). This dimer was then docked onto our previously simulated APOA1-based 80 Å HDL model (31), and the complex was subjected to 10,000 steps of energy minimization to relieve steric clashes.

Next, we compared our model to cross-linking data between APOA1 and APOA2 on native HDL isolated from plasma.

**Fig. 5:**
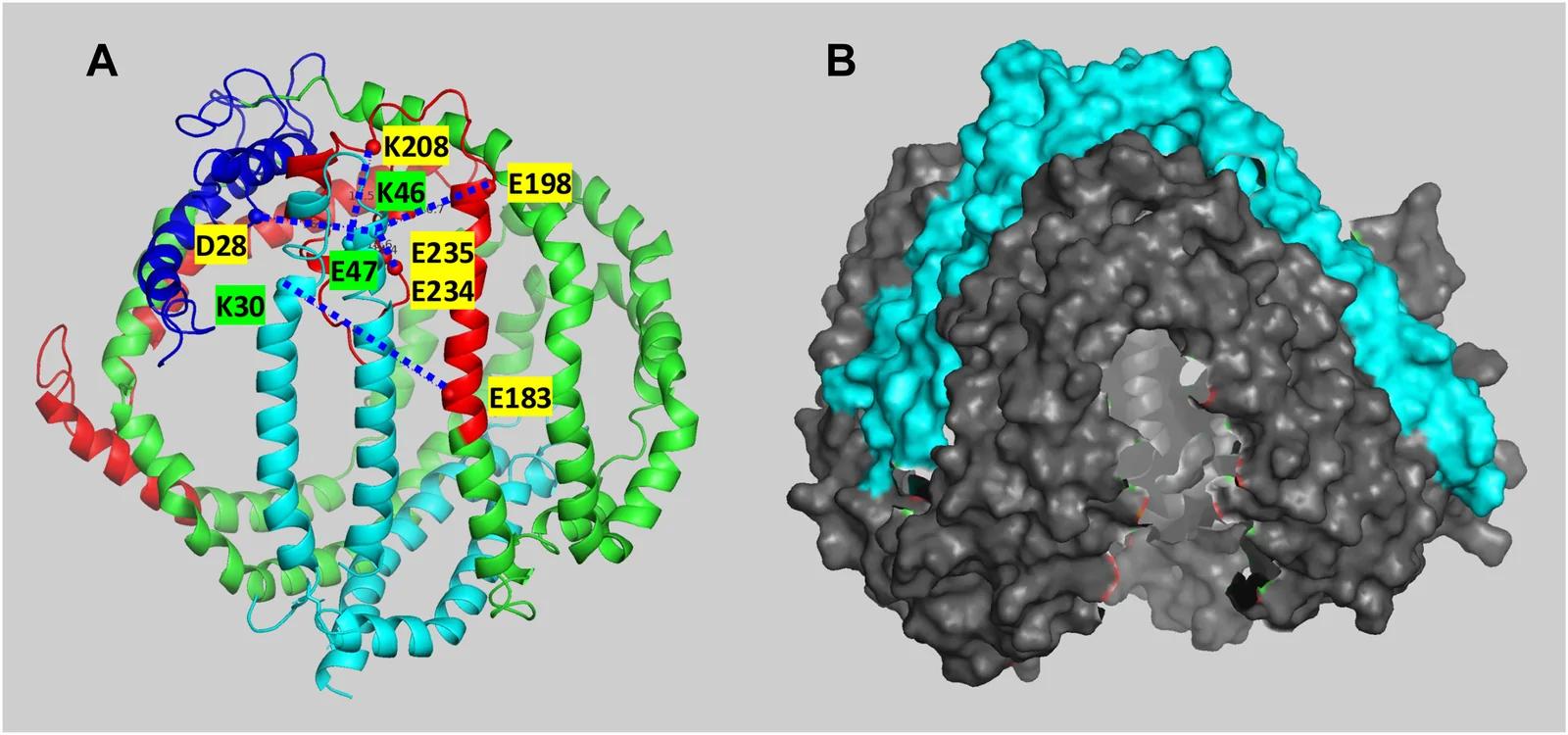
Docked model of APOA2 onto the surface of the 80 Å small spherical APOA1 particle. The APOA1 structure was taken from the all-atom model converted from the final frame of a 200 μs coarse-grained simulation reported in our previous work (31). Two potential APOA2 docking sites were identified on the small spherical APOA1 particle, and APOA2 was positioned at the site that maximized the number of satisfied experimental cross-links. The APOA2 model was then bent to accommodate the curved surface of the spherical HDL particle. A: APOA1 is shown in green, and APOA2 in cyan. The N- and C-termini of APOA1 are colored blue and red, respectively. The distances between the Cα atoms of each cross-linked residue are indicated by blue dotted lines. B: Surface representation of the model shown in panel A. APOA1 and APOA2 are colored black and cyan, respectively. Panel A was rotated to provide an improved view of the APOA1–APOA2 docking interface.

Two APOA1–APOA2 crosslinks (E234–K46: 14.4 Å; E235–K46: 12.4 Å) were ≤ 15 Å, and three crosslinks (D28–K46: 16.6 Å; E198–K46: 16.7 Å; K208–E47: 17.5 Å) fell within a reasonable 15–20 Å range. Only one crosslink (E183–K30: 24.9 Å) exceeded this range, suggesting possible local flexibility in this region of the APOA1–APOA2 interface (Fig. 5, Supplemental Table S1).

These results strongly support the proposed APOA1–APOA2 interaction model and provide structural insight into how APOA2 may engage the C-terminal region of APOA1 on small HDL particles, although more extensive molecular dynamics simulations will be needed to fully characterize the dynamics of this interaction.

## Discussion

In this study, using the LOCATE algorithm, we identified two strongly amphipathic α-helical domains (residues 5–31 and 51–73) in APOA2, separated by four prolines (P5, P32, P51, and P74). Consistent with our findings, Benetollo *et al.* also reported three amphipathic α-helical domains (residues 13–29, 34–50, and 54–70) in APOA2 (44), though their defined regions differ slightly. These differences arise from the distinct algorithms used in each study: the LOCATE algorithm identifies amphipathic α-helical domains based on termination rules (27), whereas Benetollo *et al.* employed secondary structure prediction algorithms combined with an autocorrelation method (44), which suggests helices as 17-residue-long segments. Despite these methodological differences, the overall alignment between our findings supports the robustness of our identification, with only minor variations in domain boundaries.

Our sequence alignment of APOA2 from 18 mammalian species (Supplemental Fig. S1). shows that only five species-three primates (human, gorilla, chimpanzee) and two ungulates (pig, horse)-possess a cysteine at residue 6. This cysteine in human APOA2 forms a disulfide-linked homodimer, which facilitates the formation of the HDL complex containing both APOA1 and APOA2 (LpA-I/A-II). Notably, functional studies in mice have generally reported that APOA2 promotes atherosclerosis (15, 16, 17, 18), in contrast to observations in humans. This discrepancy likely reflects differences in the structures of mouse and human APOA2. For example, in contrast to human APOA2, mouse APOA2 lacks a cysteine residue and is not disulfide-bonded in plasma. Moreover, the two proteins only share ∼60% sequence homology based on this analysis (Supplemental Fig. S1).

Interestingly, recent work by Sarkar *et al.* demonstrated that when APOA2 is present in equal mass with APOA1, it enhances ABCA1-mediated cholesterol efflux capacity compared to either protein alone, suggesting a novel functional role for APOA2 in promoting cholesterol transport and its potential as a therapeutic target for enhancing cholesterol efflux and reducing cardiovascular risk (14). Together, these findings highlight the evolutionary uniqueness of APOA2 in certain species and its potential role in cholesterol transport.

We suggest that several unique structural features of our model for HDL-associated human APOA2 are important in determining the biological effects of the binding of this second most common HDL apolipoprotein to the HDL particle surfaces: i) the extremely high lipid affinity of the fixed double belt structure of APOA2 (Fig. 1), increases the surface pressure of HDL (43) to displace specific lower lipid affinity domains of the HDL framework apolipoprotein, APOA1; ii) the flexible domain of APOA2, shown in Supplemental Fig. S4A, allows the double hairpin belt structure to curve, hydrophobic-face downward, to adjust to the highly curved surface of small HDL particles (Supplemental Fig. S4A–C); iii) the APOA2 hairpin loops containing the aromatic residues, Y41 and F42, are available to form strong π-π and π-cation bonds to certain domains of the HDL framework apolipoprotein, APOA1, helping determine APOA2’s orientation to the HDL particle surface.

In our APOA1–APOA2 model for the ∼80 Å spherical HDL particle, a disulfide-linked APOA2 homodimer (our closed double hairpin model) was docked onto the APOA1 structure derived from the final frame of a 200 μs coarse-grained simulation reported in our previous work (31) and bent to fit the spherical surface (Fig. 5). The stoichiometry of the model consists of two APOA1 molecules and one APOA2 dimer, consistent with the reported composition of LpA-I/A-II particles (34). Compared with larger HDL particles, the ∼80 Å spherical HDL has a higher surface area-to-volume ratio and greater curvature, suggesting that the APOA2 homodimer may interact more extensively with APOA1 and modify phospholipid packing. These structural features may promote lipid accessibility and cholesterol exchange, potentially accounting for the enhanced cholesterol efflux capacity often associated with small HDL particles.

Recently, using limited proteolysis and chemical cross-linking mass spectrometry, Sarkar *et al.* demonstrated that APOA2 induces displacement of the C-terminal region of APOA1 (14). Additionally, Kashiro *et al.* developed a biomarker for pancreatic cancer detection based on the APOA2 isoform ApoA2-ATQ/AT (45). Although we propose a dimeric APOA1–APOA2 model for an ∼80 Å spherical HDL particle supported by cross-linking data, our disulfide-dimer double hairpin belt model for APOA2 provides a useful blueprint for defining the molecular details of APOA2–HDL interactions in humans. Investigation of the detailed dynamics of ∼80 Å HDL particles, as well as construction of an APOA1–APOA2 model for ∼100 Å discoidal rHDL particles, is beyond the scope of the current study. Future work will investigate the effects of APOA2 on APOA1 conformation using complementary experimental approaches to validate these findings, explore detailed dynamics, and elucidate the mechanisms of APOA2 isoforms through molecular dynamics simulations.

## Data availability

All structural models generated and analyzed in this study are publicly available through Figshare at: https://doi.org/10.6084/m9.figshare.32897423.

## Supplemental data

This article contains supplemental data.

## Conflict of interest

The authors declare that they have no conflicts of interest with the contents of this article.
