# Supplementary material for: A model of human APOA2 on HDL
Source: J Lipid Res. 2026 Jul 27;67(9):101113. doi: 10.1016/j.jlr.2026.101113 (PMC13505420; doi:10.1016/j.jlr.2026.101113)
Supplement: Supplementary file 1 [file mmc1.pdf]

SUPPLEMENTAL INFORMATION:

A Model of Human APOA2 on HDL

Yi He, Hyun Song, Youngki You, John T. Melchior, Martin K. Jones, Stephen G. Aller, W. Sean Davidson, Jay W. Heinecke, and Jere P. Segrest

Crosslink Type	Residues (APOA1–APOA2)	Cα–Cα Distance (Å)
Zero-Order Crosslinks	D28-K46	16.6
	E183-K30	24.9
	E198-K46	16.7
	K208-E47	17.5
	E234-K46	14.4
	E235-K46	10.6

Supplemental Table S1. Cross-links identified between APOA1 and APOA2 using zero-length crosslinkers, with intermolecular Cα–Cα distances measured from the docked model.

```
                                         AH1              AH2
NP_001634.1    Human                QAKEPCVESLVSQYFQTVTDYGKDLMEKVKSPELQAEAKS    40
XP_004027815.1 Gorilla              QAKEPCVESLVSQYFQTVTDYGKDLMEKVKSPELQAEAKS    40
NP_001008976.1 Chimpanzee           QAKEPCVDNLVSQYFQTVTDYGKDLMEKVKSPELQAEAKS    40
XP_005541251.1 Crab-eating macaque  QAEEPSVESLVSQYFQTVTDYGKDLMEKVKSPELQAQAKA    40
NP_037244.1    Rat                  QAAETDVQTLFSQYLQSLTDYGKDLMEKAQPSEIQNQAKA    40
NP_001292478.1 Mouse                QADGPDMQSLFTQYFQSMTDYGKDLMEKAKTSEIQSQAKA    40
AAI11608.1     Cattle               QAEESNLQSLVSQYFQTVADYGKDLVEKAKGSELQTQAKA    40
XP_005677218.2 Goat                 QAEEPNLQSLVSQYFQTVADYGKDLMEKAKGSELQTQAKA    40
NP_001173223.1 Pig                  QAEEPCLQSLVSQYLQTVTDFGKDLIEKAKGSELQTQAKT    40
NP_001421632.1 Horse                QAEESCLQNLASRYLQTVTDYGKDLVEKAKAPELQAQAKA    40
XP_003415061.1 African elephant     QTEEPTLQSLFSQYLQTMSEYGKDLVEKTKAPELQAQAKA    40
XP_002715298.1 Rabbit               QAEEQSLQSLVSQYLQTVTNYSKDLMEKAKAPELQAQAKA    40
XP_007093078.1 Tiger                QAEELSLQRLVSQYFQTVTDYGKDLVEKAKGPELQAQAKA    40
XP_014931714.1 Cheetah              QAEELSLQRLVSQYFQTVTDYGKDLVEKAKGPELQAQAKA    40
XP_025842176.1 Red fox              QAEEPNLQSLVSQYFQTVTDYGKDLMEKAKGPELQAQARA    40
NP_001300728.1 Dog                  QAEEPNLQSLVSQYFQTVTDYGKDLMEKAKGPELQAQAKA    40
XP_027468347.1 California sea lion  QAEEPNLQSLVSQYFQTMTDYGKDLVEKAKGPELQAQAKA    40
XP_025716476.1 Northern fur seal    QAEEPNLQSLVSQYFQTMTDYGKDLVEKAKGPELQAQAKA    40
                                    *:    :: * ::*:*:::::.***:**.:  *:* :*::

                                      AH2             AH3
NP_001634.1    Human                YFEKSKEQLTPLIKKAGTELVNFLSYFVELGTQPATQ--     77
XP_004027815.1 Gorilla              YFEKSKEQLTPLIKKAGTELVNFLSYFVELGTQPATQ--     77
NP_001008976.1 Chimpanzee           YFEKSKEQLTPLIKKAGTELVNFLSYFMELGTQPATQ--     77
XP_005541251.1 Crab-eating macaque  YFEKSKEQLTPLVKKAGTDLVNFLSYFVELRTQPATQ--     77
NP_037244.1    Rat                  YFQNAQERLTPFVQRTGTNLMDFLSRLMSPEEKPAPAAK     79
NP_001292478.1 Mouse                YFEKTHEQLTPLVRSAGTSLVNFFSSLMNLEEKPAPAAK     79
AAI11608.1     Cattle               YFEKTQEELTPFFKKAGTDLLNFLSSFIDPKKQPATR--     77
XP_005677218.2 Goat                 YFEKTQEELTPLFKKAGTDLLNFLSSFIDPKKQPAAR--     77
NP_001173223.1 Pig                  YFEKTQEQLTPLVKKAGTDIFNFLSSFIGLKTQPTAQ--     77
NP_001421632.1 Horse                YFEKTQEQLTPLVKKIGNDLLNFFSHFIELKTQPATK--     77
XP_003415061.1 African elephant     YFEKTQEQLTPLVKKAGTDLLNFLNKFMDLKTQPAAK--     77
XP_002715298.1 Rabbit               YFEKTQEQLTPLVKKAGTDLFNFLSNFMDLKTQPAAP--     77
XP_007093078.1 Tiger                YFEKTQEQLTPLVKKAGTDLINFLNNFMDLKAQPATQ--     77
XP_014931714.1 Cheetah              YFEKTQEQLTPLVKKAGTDLINFLNNFMDLKTQPATQ--     77
XP_025842176.1 Red fox              YFEKTQEQLTPLVKKAGTDLLNFLSNFIDLKTQPATQ--     77
NP_001300728.1 Dog                  YFEKTQEQLTPLVKKAGTDLLNFLSNFMDLKTQPATQ--     77
XP_027468347.1 California sea lion  YFEKTQEQLTPLVKKAGTDLINFLSNFMDLRTQPATQ--     77
XP_025716476.1 Northern fur seal    YFEKTQEQLTPLVKKAGTDLINFLSNFMDLRTQPATQ--     77
                                    **::::*.***:.:  *..:.:*:. ::    :*:
```

Supplemental Figure S1. Sequence alignment of APOA2 in mammals. The alignment was performed using CLUSTAL Omega (version 1.2.4). The first column lists the NCBI accession numbers, and the second column indicates the corresponding species. The symbols *, :, and . indicate completely, strongly, and weakly conserved residues, respectively. AH1, AH2, and AH3 represent the three amphipathic α-helical domains in APOA2.

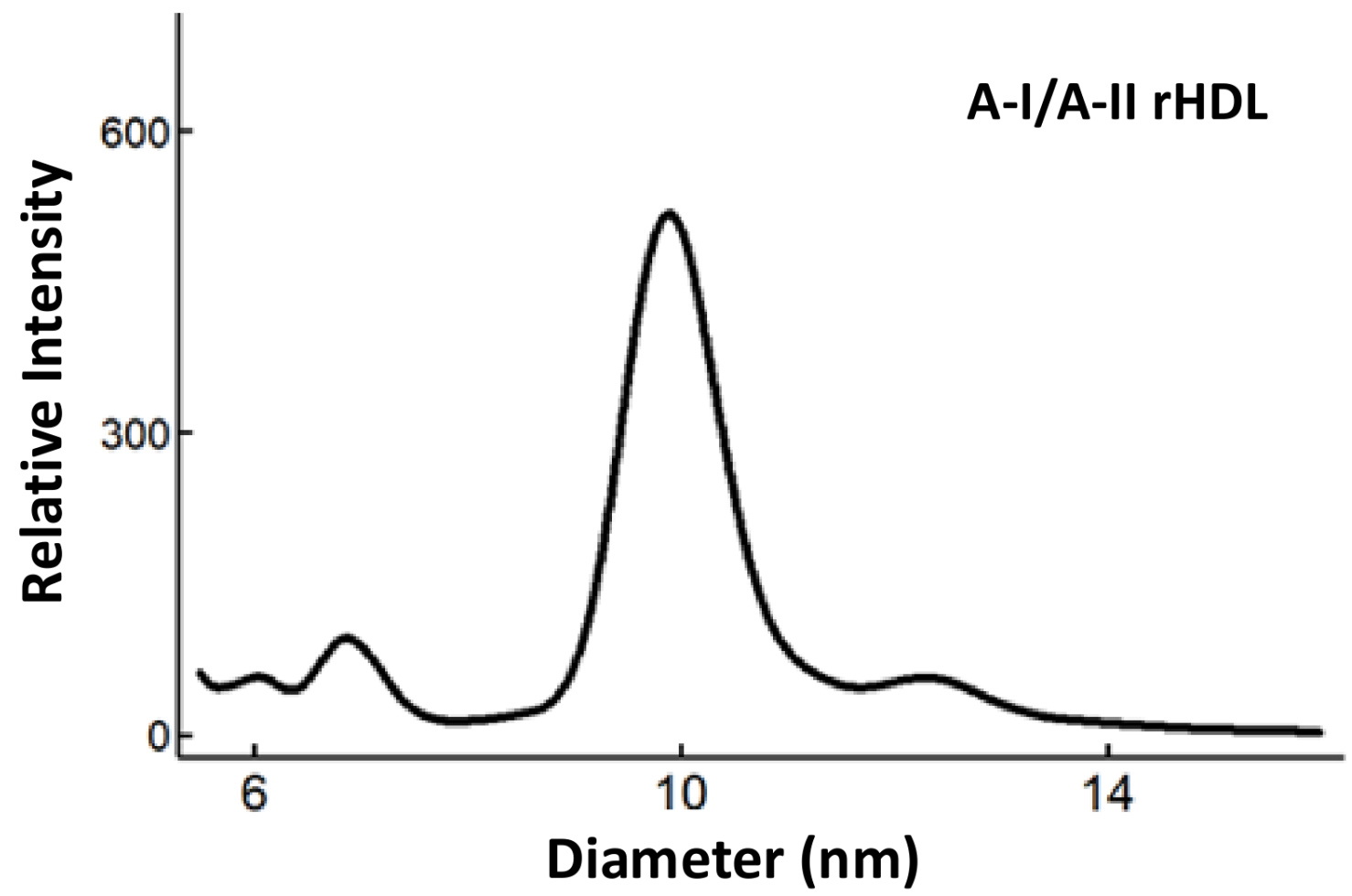

Supplemental Figure S2. The size distribution of A-I/AII rHDL. rHDL particle sizes were determined by calibrated ion mobility.

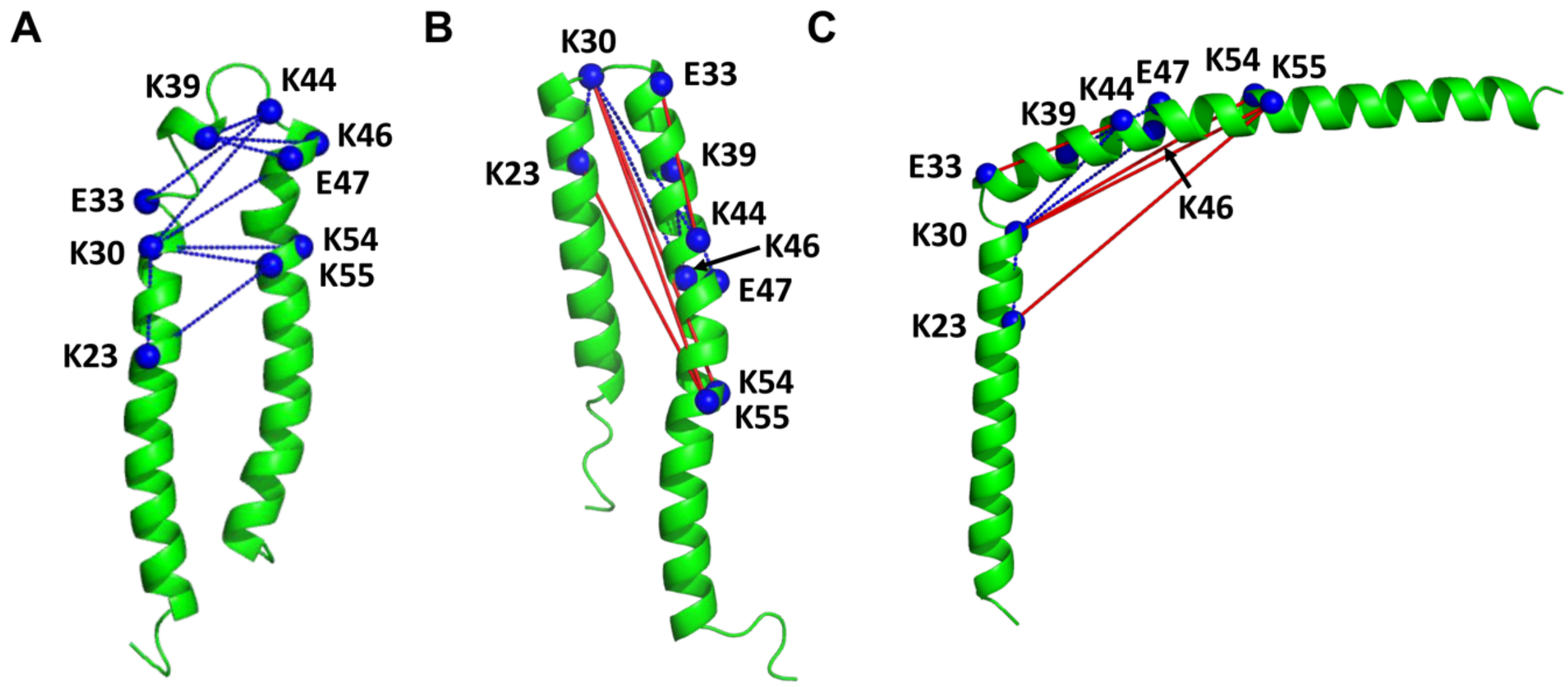

Supplemental Figure S3. Comparison of APOA2 monomer models. **A**. Our model, constructed using a combination of chemical cross-linking and computational modeling. **B**. The model generated by AlphaFold3. **C**. The model generated by RoseTTAFold2. A total of 10 intramolecular cross-link pairs from **Table 1** with strong or medium MS/MS intensities are shown. Cα atoms are represented as blue spheres. Cross-links satisfying the experimental distance cutoff (16 Å for zero-order cross-links and 25 Å for BS3/DSSO cross-links) are shown as blue dashed lines, whereas cross-links exceeding the cutoff are shown as red lines.

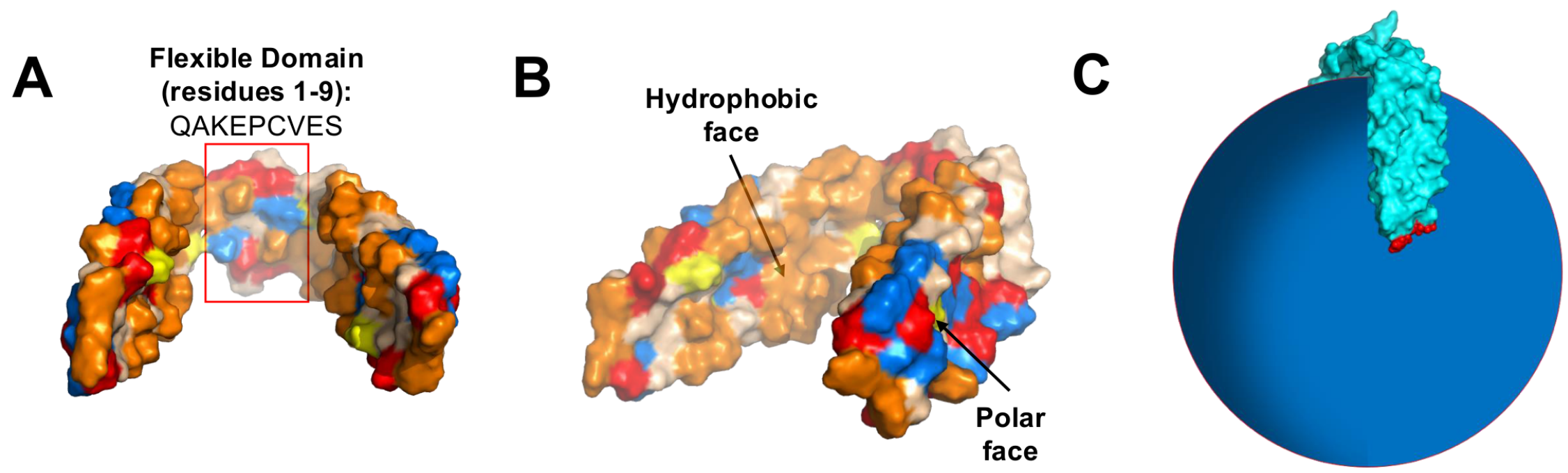

Supplemental Figure S4. Proposed modes of interaction of a disulfide-linked double hairpin belt human APOA2 with the lipid surface of HDL. A and **B**. Two views of bent double hairpin belt model of human APOA2. Basic residues, blue; acidic residues, red; hydrophobic residues, orange; prolines, yellow; other, wheat. **C**. Proposed mode of association with lipid surface of spherical HDL.
